# AI-driven multi-omics biomarkers in precision breast cancer therapeutics: from discovery to clinical translation

**DOI:** 10.3389/fphar.2026.1953714

**Published:** 2026-09-29

**Authors:** Dimitrios Stylianakis, Rodrigo Sánchez-Bayona, Nerina Denaro, Cinzia Solinas

**Affiliations:** 1 University Hospital, Heraklion, Greece; 2 School of Medicine, University of Crete, Heraklion, Greece; 3 Medical Oncology Department, Hospital Universitario 12 de Octubre, Madrid, Spain; 4 Medical Oncology Department, Hospital Universitario 12 de Octubre, Instituto de Investigación Sanitaria Hospital 12 de Octubre (imas12), Madrid, Spain; 5 Medical Oncology Unit, Fondazione IRCCS Ca’ Granda, Ospedale Maggiore Policlinico, Milan, Italy; 6 Medical Oncology Unit, University Hospital of Cagliari, Cagliari, Italy

**Keywords:** artificial intelligence, biomarkers, breast cancer, clinical translation, multiomics, precision therapeutics

## Abstract

Breast cancer is biologically heterogeneous, yet conventional clinical decisions rely on static, single-marker proxies (ER, PR, HER2, and Ki-67) that capture only a narrow slice of tumor biology. This mini-review argues that AI-driven multi-omics biomarkers offer a valuable shift toward systems-level, model-derived probabilities integrating genomic, transcriptomic, proteomic, metabolomic, and imaging information to explain and predict disease behavior more faithfully. We synthesize recent advances across three domains: (i) computational strategies for multi-omics integration, from classical penalized regression and ensemble learning to deep multimodal architectures and foundation-model embeddings; (ii) therapeutically relevant biomarker classes across tissue, liquid biopsy, and imaging modalities; and (iii) emerging pathways for embedding these biomarkers into clinical decision support. We highlight validation, interpretability, and robust handling of missing modalities as prerequisites for clinical trust, and outline how biomarker-driven trials, standardized reporting (e.g., TRIPOD + AI), and privacy-preserving data consortia can move AI-driven multi-omics biomarkers toward clinical and regulatory maturity.

## 1 Introduction

Breast cancer represents a biologically heterogeneous spectrum rather than a single disease, with outcomes diverging even among tumors sharing the same receptor-defined classification (Turner et al., 2021; Prat et al., 2015). This heterogeneity is particularly evident in triple-negative breast cancer (TNBC), which, despite its clinical recognition, encompasses multiple biologically distinct entities and still lacks broadly effective tumor-specific targeted therapies (Zagami and Carey, 2022). Among hormone receptor-positive tumors, Luminal A and Luminal B subtypes likewise differ in proliferation, prognosis, and treatment sensitivity, while HER2-positive disease includes both highly targetable and more treatment-refractory phenotypes (Rios-Hoyo et al., 2025; Pernas and Tolaney, 2020). In metastatic disease, treatment decisions remain largely guided by conventional staging and primary-tumor receptor status, despite evidence that metastatic sites have distinct biological and therapeutic characteristics (Yeung et al., 2016). Bone, visceral, and central nervous system metastases therefore present different prognostic and treatment challenges that may not be fully captured by the biology of the primary tumor (Venetis et al., 2021; Morgan et al., 2021). Together, these limitations highlight the need for biomarkers that better reflect the molecular complexity of breast cancer across subtypes and sites of disease.

Current biomarkers are insufficient for this complexity. Ki-67, a nuclear marker of proliferation whose dynamic measurement can assess treatment response, remains widely used, yet inter-laboratory variability and inconsistent scoring thresholds continue to undermine its reproducibility and clinical reliability (Gown et al., 2023). Likewise, single-marker frameworks centered on estrogen receptor (ER), progesterone receptor (PR), human epidermal growth factor receptor 2 (HER2), and Ki-67 capture only a narrow slice of tumor biology, overlooking the coordinated genomic, transcriptomic, proteomic, and metabolomic programs, as well as immune and stromal interactions, that shape progression and therapeutic response. Gene-expression profiles, approximated clinically by ER, PR, HER2, and Ki-67, underpin the major intrinsic molecular subtypes: luminal A, luminal B, HER2-enriched, and basal-like, which have distinct prognostic and therapeutic implications (Prat et al., 2015; Cardoso et al., 2019). Single-cell and spatial profiling have made this limitation increasingly apparent by revealing levels of intratumoral heterogeneity that bulk, single-omics approaches cannot resolve (Xiong et al., 2024; Stylianakis et al., 2026). Throughout this review, we use the term ‘intrinsic subtypes’ to refer to these receptor- and expression-based classes.

Artificial intelligence offers the integrative framework needed to move beyond these constraints (Figure 1). AI-driven multi-omics biomarkers combine two or more molecular or imaging modalities within machine learning or deep learning models to identify shared and complementary patterns across data types. This approach is already reshaping oncology, from foundation model embeddings that unify pathology, radiology, and molecular data into patient-level representations (Menon et al., 2026) to multimodal frameworks such as HONeYBEE (Tripathi et al., 2025), clinical-grade pathology models for rare cancer detection (Vorontsov et al., 2024), and broader efforts in precision oncology and multi-omics integration (Truhn et al., 2024; Muneer et al., 2025; Yates and Van Allen, 2025).

**FIGURE 1:**
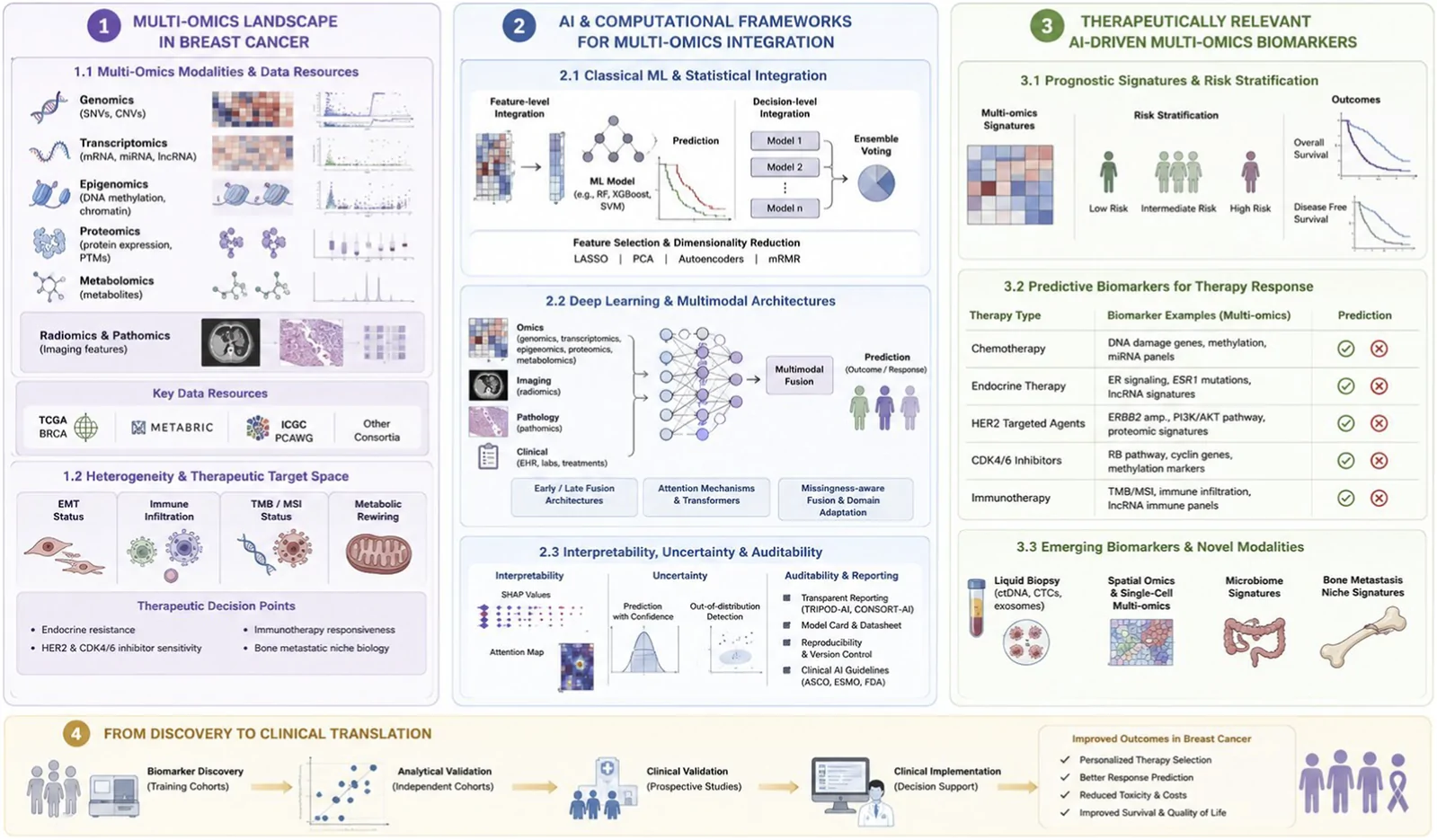
Conceptual framework of AI-driven multi-omics biomarker discovery and clinical translation in precision breast cancer therapeutics. Integration of molecular and imaging data through machine learning and deep learning enables biomarker discovery for prognostic stratification, treatment response prediction, metastasis assessment, and disease monitoring. The framework illustrates the translational continuum from multi-omics data integration and model development to biomarker validation, clinical implementation, and precision oncology.

This mini narrative review examines how these integrative AI approaches are being applied in breast cancer to develop biomarkers that support risk stratification, therapy selection, response prediction, and resistance monitoring.

## 2 Multi-omics landscape in breast cancer

### 2.1 Multi-omics modalities and data resources

Breast cancer research increasingly integrates genomic, transcriptomic, epigenomic, proteomic, and metabolomic layers for a more complete view of tumor biology than any single platform allows. The Molecular Taxonomy of Breast Cancer International Consortium (METABRIC) linked copy-number and expression profiles from nearly 2,000 tumors to long-term outcomes, revealing molecular subgroups beyond standard receptor-based classification (Curtis et al., 2012). Multi-omics factor analysis of an independent cohort identified three prognostic clusters validated in METABRIC and The Cancer Genome Atlas (TCGA), outperforming intrinsic subtyping for survival prediction beyond 12 years (Sharma et al., 2024).

Network- and attention-based models applied to copy-number, expression, and methylation data have refined subtype boundaries, uncovering hybrid phenotypes, such as a poor-prognosis subtype marked by low immune infiltration and T-cell dysfunction, outside the luminal A/B, HER2-enriched, and triple-negative framework (Wang et al., 2023; Choi and Chae, 2023). Radiomics and pathomics have emerged as imaging-derived, “omics-like” data types: quantitative MRI or digitized histopathology features can be modeled alongside genomic data, and combined radio-pathomic models have predicted pathological response to neoadjuvant chemotherapy more accurately than either modality alone (Xu et al., 2024; Qi et al., 2024).

### 2.2 Biological heterogeneity and therapeutic target space

Multi-omics studies map heterogeneity axes shaping clinical behavior beyond histological subtype: epithelial–mesenchymal transition (EMT) activity, immune infiltration, tumor mutational burden, and metabolic reprogramming. Enhancer-level analyses have linked copy-number-driven regulatory elements to divergent prognoses within luminal and basal-like tumors, showing canonical subtypes conceal further stratification (Zhao H. et al., 2022). Individual-level integration has extended this mapping to single patients, addressing batch effects limiting clinical transferability of group-level subtyping (Zhao Z. et al., 2023).

These axes intersect directly with treatment choice: immune-infiltrated, high-mutational-burden tumors favor checkpoint inhibition, growth-factor-driven luminal signaling motivates CDK4/6 or PI3K-pathway inhibition, and EMT-associated programs drive resistance and metastasis (Ran et al., 2025). Because these features interact non-additively, modeling their combined effect on therapy response suits AI-based multi-omics integration better than conventional low-dimensional statistics (Correa-Aguila et al., 2022).

## 3 AI and computational frameworks for multi-omics integration

In this section, we briefly outline the main computational approaches used for multi-omics integration. Classical machine learning (ML) models, such as penalized regression and tree-based ensembles, rely on predefined features, whereas deep learning methods learn multi-layer representations directly from raw data. Autoencoders are neural networks trained to compress and reconstruct inputs, enabling dimensionality reduction and feature extraction, while attention-based graph convolutional networks operate on graph-structured data, weighting more informative connections between samples or genes. Deep latent-variable models learn shared, low-dimensional representations (‘latent variables’) that capture structure across multiple data types.

### 3.1 Classical ML and statistical integration approaches

Classical machine-learning methods remain the workhorse of multi-omics modeling in breast cancer. Regularized regression, random forests, gradient boosting, and support-vector classifiers are commonly used for decision- or feature-level integration: decision-level approaches train separate models per omics layer and pool outputs, whereas feature-level approaches concatenate selected features across modalities into a single input matrix. For example, survival models integrating DNA methylation and miRNA features through complementary and consensus autoencoder architectures improved predictive concordance from the 0.58–0.62 range for single-omics models to approximately 0.64 after multi-omics integration in TCGA BRCA (Tong et al., 2020). Similar feature-level approaches have combined multiple molecular layers to improve survival prediction, while feature selection and dimensionality reduction help prioritize informative, non-redundant features in high-dimensional multi-omics matrices (Correa-Aguila et al., 2022). Attention-based graph convolutional networks integrating methylation, copy-number, and expression data have also improved subtype classification over single-omics baselines while flagging patient-specific driver genes (Guo et al., 2023), and moBRCA-net achieved comparable gains by learning omics-level importance weights rather than treating all layers equally (Choi and Chae, 2023).

### 3.2 Deep learning and multimodal architectures

Deep learning extends multi-omics integration beyond fixed feature concatenation toward architectures that learn shared and modality-specific representations directly from data. Attention-based convolutional networks applied to image-transformed multi-omics inputs have improved subtype classification and prognostic gene discovery relative to earlier feature-engineering approaches (Li et al., 2026), while deep latent-variable models designed to fuse imaging, omics, and clinical data into a shared representation space are being generalized across cancer types, including breast cancer (Ing et al., 2025).

Multimodal fusion of omics-adjacent imaging with clinical variables has reached outcome-level validation. A model integrating whole-slide histopathology, ultrasound imaging, and structured clinical data achieved a concordance index of 0.84 for recurrence prediction in hormone receptor-positive, HER2-negative early breast cancer, exceeding single-modality comparators (Wu X. et al., 2026). Earlier work combining histopathology images with clinical variables for HER2-positive disease similarly improved recurrence and metastasis prediction, replicating in an independent TCGA cohort despite differing patient populations and assay platforms (Yang et al., 2021).

A persistent obstacle is structural missingness: most patients lack complete multi-omics or multimodal profiles in routine care. Reviews of integration methods emphasize that robust deployment requires tolerance for partial data and platform variability rather than models assuming uniformly complete inputs—an issue only partially resolved in current architectures (Correa-Aguila et al., 2022; Hsu et al., 2025).

### 3.3 Interpretability, uncertainty, and model auditability

Interpretability is necessary to translate multi-omics outputs into credible biological hypotheses rather than opaque risk scores. Attention-weighted architectures such as moBRCA-net and attention-based graph networks directly expose which omics layers and features drive individual predictions, supporting biological interpretation without separate *post hoc* explanation methods (Choi and Chae, 2023; Guo et al., 2023).

Uncertainty quantification addresses a related but distinct problem: knowing when a prediction should not be trusted. In digital histopathology, dropout-based uncertainty estimation flags a substantial proportion of cases a model is likely to misclassify, allowing low-confidence predictions to be routed for expert review rather than acted upon automatically (Dolezal et al., 2022; Linmans et al., 2023). Extending such calibrated, uncertainty-aware frameworks to multi-omics breast cancer models, together with transparent reporting of training data, validation cohorts, and failure modes, will likely be a prerequisite for regulatory acceptance and clinician trust. However, this integration has not yet been widely demonstrated in multi-omics-specific architectures.

## 4 Therapeutically relevant biomarker classes across modalities

In this section, we organise AI-driven multi-omics biomarkers by clinical application: prognostic and predictive risk stratification, metastatic propensity, and longitudinal monitoring, while explicitly indicating whether they are tissue-based, liquid biopsy-derived, or imaging-integrated.

### 4.1 Tissue-based prognostic and predictive signatures

#### 4.1.1 Prognostic signatures and risk stratification

Multi-omics frameworks have produced prognostic signatures that move beyond single-gene or receptor-based models (Table 1). A six-gene signature from integrated RNA-seq, single nucleotide polymorphism (SNP), and copy-number data in TCGA BRCA used random forest selection and Cox modeling, achieving 5-year AUCs above 0.65 and retaining prognostic value across GEO cohorts (Mo et al., 2020). In TNBC, a multi-omics pipeline integrating methylation and transcriptomic data identified a 47-gene prognostic panel with cross-platform generalizability from TCGA RNA-seq to an independent microarray cohort, achieving ∼75% external accuracy with biologically coherent Shapley additive explanations (SHAP) explanations (Nagendra and Natrajan, 2026, preprint).

**TABLE 1:** AI-driven multi-omics biomarker classes in breast cancer: modalities, algorithms, evidence, and translational readiness.

Biomarker class	Key evidence	Omics/data layers integrated	Representative AI/ML approaches	Primary clinical application	Typical validation stage	Translational readiness	Key limitations
Integrated prognostic signatures	Multi-omics clustering identified long-term prognostic groups that outperformed intrinsic subtypes for long-horizon survival stratification (Sharma et al., 2024). A TCGA-derived 6-gene integrated signature was externally tested in GEO and remained prognostic across subtype analyses (Mo et al., 2020)	Genomics, CNV, transcriptomics, DNA methylation; sometimes proteomics and metabolomics	LASSO-Cox, random forest, gradient boosting, multi-kernel clustering, autoencoders	Long-term risk stratification; refinement of recurrence risk within receptor-defined disease	Discovery in TCGA/Oslo2; retrospective external validation in METABRIC or GEO	Moderate for research use; low for routine clinical deployment	Batch effects, assay transferability, modest gain over clinicopathologic models, lack of prospective utility studies
Immune- and lncRNA-based prognostic models	Immune-related lncRNA signatures were associated with survival, immune infiltration, and tumor mutational burden in breast cancer (Huang et al., 2021; Ma et al., 2020). Pyroptosis-immune and disulfidptosis-linked lncRNA signatures further connected risk strata with checkpoint biology and putative immunotherapy sensitivity (Kong et al., 2025; Zhou et al., 2025)	lncRNA expression, immune gene signatures, transcriptomics, TMB-linked features	Cox modeling with penalized selection, random forest ranking, enrichment-based feature integration	Prognosis; immune-context stratification; hypothesis generation for immunotherapy selection	Retrospective, multi-cohort validation	Moderate as exploratory biomarkers; low as clinical assays	Biological interpretability varies; lncRNA assays are not standardized; clinical added value versus simpler immune markers remains uncertain
Stemness/metabolism-linked risk models	A stemness-associated multi-omics model defined molecular subtypes with distinct prognosis and immune depletion, supporting stemness as a clinically relevant axis of stratification (Wang et al., 2023). Mitochondrial and metabolic lncRNA signatures similarly linked prognosis to immune microenvironment differences (Huang et al., 2021)	Transcriptomics, stemness indices, metabolic programs, immune features	Consensus clustering, risk-score modeling, feature selection pipelines	Identification of biologically aggressive states not captured by standard subtype labels	Discovery with external retrospective validation	Emerging	Limited harmonization of stemness metrics; difficult assay simplification; unclear therapeutic actionability in routine care
Predictive therapy-response biomarkers	Reviews and integrative studies indicate that multi-omics AI models can improve prediction of treatment response and resistance by combining molecular and clinical layers rather than relying on single biomarkers alone (Hsu et al., 2025; Ran et al., 2025). TNBC multi-omics studies have also linked integrated subtypes to candidate therapeutic vulnerabilities (Lehmann et al., 2021)	Genomics, transcriptomics, epigenomics, proteomics, metabolomics, clinical variables	Ensemble learning, multi-task models, deep multimodal fusion, attention-based networks	Prediction of chemotherapy, endocrine therapy, HER2-targeted therapy, CDK4/6 inhibitor, and immunotherapy response	Mostly retrospective; limited clinical-trial embedding	Moderate for translational research; low-to-moderate clinically	Endpoint heterogeneity, treatment-era drift, incomplete modalities, limited decision-impact evidence
Radiomics-integrated response biomarkers	Multicohort radiomic analyses linked imaging-derived response patterns after neoadjuvant chemotherapy with survival and molecular programs (Fan et al., 2024). PET radiomics studies showed that combined intratumoral and peritumoral features improved prediction of pathological response (Aksu et al., 2024)	MRI, PET/CT, mammographic or pathological image features plus molecular signatures or clinicopathologic variables	CNNs, 3D deep learning, multimodal fusion, radiogenomic mapping	Neoadjuvant response prediction; recurrence-risk refinement; imaging surrogates of molecular behavior	Retrospective multicohort studies	Moderate in specialized centers; low as standardized multi-omics tools	Imaging protocol variability, unstable radiomic pipelines, limited cross-site calibration, difficult biological interpretability
Metastatic propensity/organotropism biomarkers	A machine-learning multi-omics framework identified PRDX4 as a redox-EMT regulator and predictive marker in bone-metastatic breast cancer, linking molecular programs to metastatic behavior (Zhou et al., 2026). Metastasis-related gene signatures have also shown prognostic value across discovery and validation cohorts (Wang ZZ et al., 2023)	Transcriptomics, proteomics, phosphoproteomics, EMT-related programs, immune/stromal features	Network-based ML, clustering, LASSO-Cox, multi-omics module analysis	Prediction of metastatic risk, bone tropism, and microenvironment-linked vulnerability	Discovery and preclinical/retrospective validation	Emerging	Site-specific cohort bias, limited prospective validation, complex assays, uncertain pathway from signature to deployable test
Microenvironment-focused biomarkers	Immune-molecular clustering in TNBC models identified subgroups with distinct chemo-immunotherapy sensitivity, illustrating that therapy response may depend on both tumor-intrinsic state and immune composition (Castellanet et al., 2026). Cancer-associated fibroblast-linked lncRNA signatures further support stromal contributions to prognosis (Wang ZZ et al., 2023)	Immune cell signatures, stromal programs, lncRNAs, transcriptomics, occasionally spatially informed features	Consensus clustering, pathway enrichment, subtype learning, network inference	Identification of immune-inflamed, immune-excluded, or stromal-dominant states with therapeutic relevance	Preclinical or retrospective translational studies	Emerging	Spatial heterogeneity, assay complexity, limited prospective linkage to treatment decisions
Liquid biopsy multi-omics biomarkers	Reviews of breast cancer liquid biopsy emphasize the translational potential of combining ctDNA, CTCs, exosomal RNA, and extracellular vesicle cargo for MRD detection, relapse prediction, and resistance monitoring (Simancas-Racines et al., 2025; Qiu et al., 2025). Exosome-centered multi-OMIC reviews support EV-based multi-analyte biomarker development (Ansermino et al., 2026)	ctDNA mutation/CNV/methylation, CTC phenotype, exosomal RNA, EV proteomics/metabolomics	Multi-analyte fusion models, regularized regression, ensemble classifiers, deep learning on cfDNA-derived features	MRD detection, relapse prediction, dynamic monitoring of clonal evolution and resistance	Analytic validation for some ctDNA assays; broader multi-omic panels remain translational	High near-term relevance; moderate current readiness	Pre-analytical variability, sensitivity thresholds, cost, unclear action thresholds, need for inter-laboratory standardization
Imaging-genomics/multimodal patient-representation biomarkers	Multimodal oncology foundation models and embedding frameworks have shown that pathology, radiology, and molecular data can be integrated into unified patient-level representations, with potential value for stratification and clinical support (Menon et al., 2026; Tripathi et al., 2025)	Radiology, pathology, molecular profiles, structured clinical data	Foundation-model embeddings, multimodal transformers, joint latent-space learning	Cross-modal risk prediction, subtype inference, decision support	Early translational/platform-level development	Emerging but strategically important	Limited breast cancer-specific prospective evidence, interpretability concerns, infrastructure burden, regulatory immaturity
Biomarker-guided nanomedicine and delivery strategies	AI-assisted and biomarker-driven nanomedicine frameworks have been proposed to align delivery platforms with molecular and liquid-biopsy-defined targets in breast cancer, but evidence remains largely preclinical (Barar et al., 2026)	Tissue biomarkers, liquid biopsy signals, pharmacogenomic features, payload-delivery parameters	Optimization algorithms, supervised learning, AI-assisted formulation design	Precision drug delivery and patient selection for targeted nanotherapeutics	Preclinical and early-phase development	Conceptually promising; clinically early	Regulatory complexity, co-development of companion diagnostics, manufacturing burden, limited human validation

The examples in Table 1 were identified through a narrative synthesis of representative, clinically relevant cases rather than an exhaustive systematic review. Translational readiness categories reflect the authors’ assessment based on evidence of external validation, assay robustness, and incorporation into prospective studies or clinical workflows. In this context, ‘High’ indicates analytic validation and emerging clinical use (for example, ctDNA MRD assays in recurrence monitoring), ‘Moderate’ indicates strong retrospective data but limited trial embedding, and ‘Emerging’ denotes promising preclinical or early translational work without direct clinical implementation. Abbreviations: AI, artificial intelligence; CDK4/6, cyclin-dependent kinases four and 6; cfDNA, cell-free DNA; CIMLR, cancer integration *via* multikernel learning; CNV, copy-number variation; CNN, convolutional neural network; CTC, circulating tumor cell; ctDNA, circulating tumor DNA; EMT, epithelial-mesenchymal transition; EV, extracellular vesicle; GEO, gene expression omnibus; HER2, human epidermal growth factor receptor 2; HR, hormone receptor; lncRNA, long non-coding RNA; ML, machine learning; MRD, minimal residual disease; MRI, magnetic resonance imaging; PET, positron emission tomography; TMB, tumor mutational burden; TCGA, the cancer genome atlas; TNBC, triple-negative breast cancer.

Immune-related lncRNA signatures provide complementary stratification, separating patients into risk groups with consistent survival differences and ROC AUCs of ∼0.68–0.71 in external validation (Huang et al., 2021; Ma et al., 2020). Mechanistically focused panels (pyroptosis-immune, disulfidptosis-related, necroptosis-related, and mitochondrial-function-related lncRNAs) reveal distinct immune infiltration, checkpoint expression, and metabolic states co-segregating with prognosis (Kong et al., 2025; Wang et al., 2023; Yu et al., 2023; Chen et al., 2022), with high-risk groups often showing elevated tumor mutational burden and altered immune scores, bridging prognostic and predictive biomarker roles.

Generalizability remains a central challenge: many signatures rely on TCGA RNA-seq discovery with only modest transfer testing to microarray, single-cell, or clinically curated cohorts, where batch effects degrade performance (Mo et al., 2020; Huang et al., 2021). Robust adoption requires harmonized pipelines and multi-center validation demonstrating added clinical discrimination beyond established models.

#### 4.1.2 Predictive biomarkers for therapy response

AI-multi-omics approaches increasingly target therapy response and resistance. An integrative framework combining genomic, transcriptomic, proteomic, and clinical variables stratified endocrine and chemotherapy resistance, identifying signatures predicting non-response to anthracyclines and endocrine therapy (Ran et al., 2025). Multi-omics profiling in TNBC uncovered clusters with differential sensitivity to EGFR, PARP, and mTOR inhibitors, mapping chemo-targeted vulnerabilities missed by single-layer biomarkers (Lehmann et al., 2021; Jovanović et al., 2024).

Mechanism-focused lncRNA signatures link risk scores to therapy response: disulfidptosis-related panels show differential predicted sensitivity to chemotherapy and checkpoint inhibitors (Zhou et al., 2025); necroptosis-related signatures associate higher risk with elevated tumor mutational burden and lower Tumor Immune Dysfunction and Exclusion (TIDE) scores, implying better PD-1/PD-L1 responsiveness (Chen et al., 2022); and senescence-related lncRNA models stratify immunotherapy response and drug susceptibility *via* integrated mutational and immune infiltration data (Kong et al., 2025).

Radiomics-genomics integration adds a non-invasive dimension: multicohort radiomic analysis of NAC identified imaging-based response patterns correlating with survival and molecular programs (Fan et al., 2024), while PET radiomics combining intratumoral and peritumoral features predicted pathological complete response more accurately than standard imaging, linkable to immune and proliferation pathways (Aksu et al., 2024). Reviews emphasize that combining imaging with genomic/transcriptomic profiles improves prediction of subtype, mutation status, and immunotherapy/targeted response, though evidence remains largely retrospective (Wu and Dai, 2025; Ran et al., 2025).

### 4.2 Metastatic propensity and microenvironment biomarkers

Multi-omics frameworks are dissecting metastatic risk and organotropism, particularly in bone-metastatic disease. An integrative ML platform combining EMT- and nucleotide-metabolism (NM) signatures identified PRDX4 as a redox-EMT regulator and predictive marker of skeletal relapse (Zhou et al., 2026), while phosphoproteomic analyses highlighted dysregulated kinases (PRKDC and MAP4K4) cooperating with EMT programs in basal-like clusters (Lei et al., 2023).

Microenvironment-focused studies reveal parallel axes of metastatic propensity: multi-omics profiling in a TNBC mouse model identified four immune-molecular clusters with distinct chemo-immunotherapeutic vulnerabilities: neutrophil-enriched tumors responded better to epirubicin plus anti-PD-1, versus macrophage-enriched tumors favoring immunotherapy alone—showing organotropism and therapy response are jointly shaped by EMT state and immune composition (Castellanet et al., 2026). These biomarkers are not yet clinically approved tests but delineate candidate hubs like PRDX4 and specific kinase/immune modules for precision intervention.

AI-guided *in silico* screening and docking workflows are increasingly layered onto such discoveries, using network-inferred hubs to prioritize small molecules or repurposed cytotoxics for preclinical testing (Hsu et al., 2025; Gouru et al., 2025). Most claims remain preclinical, but they illustrate how metastasis-focused multi-omics biomarkers can feed directly into rational drug design and repurposing.

One illustrative TNBC example used multi-omics integration of genomes, transcriptomics, proteomics, and metabolomics to define four subtypes (basal-like 1 (BL1), basal-like 2 (BL2), mesenchymal, and luminal androgen receptor (LAR)) each enriched for distinct dysregulated pathways such as DNA damage response, immune modulation, or growth-factor signaling (Lehmann et al., 2021). Overlaying these subtype-specific networks with drug-target databases identified vulnerabilities to PARP inhibition in DNA repair-deficient clusters and EGFR or mTOR pathway targeting in growth-factor-driven clusters, providing concrete hypotheses for rationally designed, subtype-specific combination regimens. Subsequent work has refined these insights using expanded multi-omics datasets, converging on overlapping TNBC biomarkers and pathways that inform clinical trial design (Jovanović et al., 2024).

In bone-metastatic breast cancer, integrative multi-omics and machine-learning analyses highlighted PRDX4 as a candidate therapeutic target by showing that its redox-EMT regulatory role was conserved across transcriptomic, proteomic, and phosphoproteomic layers, and functionally linked to skeletal relapse (Zhou et al., 2026). Mechanistic validation in experimental models demonstrated that perturbing PRDX4 or associated pathways modulated EMT markers and metastatic behaviour, supporting its candidacy for targeted or combination interventions. Together, these vignettes show a coherent translational logic: multi-omics-derived biomarkers identify mechanistic hubs, *in silico* prioritization proposes drug targets, and preclinical validation tests those hypotheses, forming a template for future biomarker-to-therapy pipelines (Hsu et al., 2025).

### 4.3 Liquid biopsy-based multi-omics biomarkers

Liquid biopsy components, including circulating tumor DNA (ctDNA), circulating tumor cells (CTCs), exosomal RNA, and extracellular-vesicle (EV) proteomic and metabolomic cargo, offer a minimally invasive window into breast cancer biology. Reviews describe ctDNA mutational and methylation signatures being evaluated for early detection, minimal residual disease (MRD) monitoring, and tracking emergent resistance, while CTC enumeration and PD-L1 expression provide prognostic and immunotherapy-relevant information (Qiu et al., 2025; Ghanim Taki et al., 2026). Multi-omics integration of these analytes has yielded composite panels that more accurately reflect tumor burden and clonal architecture than single liquid biopsy metrics alone (Simancas-Racines et al., 2025).

AI-driven models built on liquid biopsy multi-omics are beginning to support longitudinal decision-making, merging cfDNA methylation, fragmentomics, and other liquid-derived omics into predictive frameworks for dynamic risk stratification, though most remain proof-of-concept (Ran et al., 2025; Hsu et al., 2025). Clinical evidence for MRD and relapse prediction is emerging: prospective studies correlate post-therapy ctDNA detection with early relapse, and trials are testing ctDNA-guided escalation/de-escalation versus standard imaging follow-up (Qiu et al., 2025; Simancas-Racines et al., 2025). Standardization, low-abundance variant detection, and cost remain major barriers; nevertheless, the integration of longitudinal liquid-biopsy data with artificial intelligence offers a plausible route to adaptive therapeutic decision-making, exemplified by emerging efforts to couple serial ctDNA-based Estrogen Receptor one monitoring with computational analysis of endocrine-resistance evolution in ER-positive disease (Martella et al., 2025).

### 4.4 Imaging-genomics and radiomics-integrated biomarkers

AI methods fusing radiomics with genomic data expand the biomarker space into multimodal territory. Reviews describe models combining mammography, MRI, or PET/CT features with gene-expression and mutation profiles to predict molecular subtypes, driver alterations, and treatment response (Wu and Dai, 2025). Multicohort radiomic analyses of NAC show imaging-derived response patterns (textural, shape, and peritumoral features) correlate with survival and map to proliferative and immune pathways, functioning as imaging surrogates of treatment-induced trajectories (Fan et al., 2024).

AI-assisted breast imaging offers complementary clinical evidence: a large AI-based computer-aided detection (AI-CAD) study found that integrating AI-derived outputs into subspecialist workflows increased cancer detection rate by roughly 14% without inflating recall rates, although broader evidence remains limited (Ran et al., 2025; Stylianakis et al., 2026). Frameworks combining digital pathology, spatial transcriptomics, and multi-omics with radiomics are being explored to capture spatially resolved tumor–microenvironment interactions (Ing et al., 2025; Kim et al., 2026), outlining a plausible path toward unified multi-omics-plus-imaging systems for therapy selection and monitoring.

## 5 Translational pipelines, validation, and regulatory considerations

### 5.1 From discovery to clinical assay: validation and standardization

Most AI-driven multi-omics biomarkers start with retrospective discovery in TCGA, METABRIC, or institutional cohorts, followed by internal validation *via* cross-validation or held-out sets (Hsu et al., 2025; Sabit et al., 2026). Subsequent steps include external validation in independent cohorts and assay optimization—translating signatures into usable panels *via* targeted sequencing or multiplexed proteomics (Yasmin et al., 2025). Only once analytic performance and clinical utility are demonstrated can biomarkers enter prospective trials as stratification factors or companion diagnostics (Sabit et al., 2026).

Assay standardization is a recurrent bottleneck: platform harmonization, batch-effect correction, and pre-analytical variability erode reproducibility (Yasmin et al., 2025; Simancas-Racines et al., 2025). Without shared reference standards and preprocessing pipelines, multi-center validation yields inconsistent estimates (Jiang et al., 2025). Prospective, multi-center studies prespecifying biomarker assays and trial endpoints are critical to moving these tools from discovery to clinically approved tests (Yasmin et al., 2025; Sabit et al., 2026).

### 5.2 Clinical workflow integration and decision support

Embedding AI-multi-omics biomarkers into workflows requires more than discrimination metrics (Table 1). Clinician-focused guides outline decision-support interfaces linked to electronic health records and tumor boards, with visualizations breaking down feature and pathway contributions (Hsu et al., 2025; Wang et al., 2025). Practical constraints, like uneven modality availability, turnaround times, infrastructure, and cost, may favor stepwise adoption starting with lower-complexity liquid biopsy panels (Sabit et al., 2026). A few institutions are piloting integrated genomic-radiomic-AI pipelines in tumor boards, but these remain early exemplars (Hsu et al., 2025).

### 5.3 Ethical, regulatory, and equity considerations

Ethical analyses emphasize data privacy, informed consent, and algorithmic bias from underrepresentative training data (Correa-Aguila et al., 2022; Bonetti et al., 2023). Regulatory guidance expects transparent reporting and post-market surveillance, with TRIPOD + AI and PROBAST-AI providing structure for documenting model development and limitations (Collins et al., 2024; Collins et al., 2021). Without attention to affordability and infrastructure (including federated learning and cost-efficient assays) multi-omics AI risks widening global diagnostic disparities (Sabit et al., 2026).

## 6 Discussion

AI-driven multi-omics biomarkers mark a shift from static, single-marker paradigms- ER, PR, HER2, Ki-67 - toward systems-level, model-derived probabilities integrating genomic, transcriptomic, proteomic, metabolomic, and imaging features (Hsu et al., 2025; Yasmin et al., 2025). This reflects that breast cancer behaviour emerges from coordinated pathway activity and microenvironmental context, requiring models capable of learning non-linear, cross-modal relationships (Zhang et al., 2024).

The main translational barrier is not algorithmic but validation-related (Table 1). Many signatures are derived in single large cohorts (TCGA or METABRIC) and only modestly tested externally, with limited clinical annotation (Mo et al., 2020; Sharma et al., 2024), making overfitting a real risk (Yasmin et al., 2025). Interpretability is another prerequisite: attention-based networks exposing omics- and feature-level importance, alongside SHAP and pathway analyses, help clinicians see what drives a recommendation (Choi and Chae, 2023; Guo et al., 2023). Uncertainty-aware deep learning in histopathology shows calibrated estimates can flag likely misclassifications for manual review, a strategy adaptable to multi-omics contexts (Dolezal et al., 2022; Linmans et al., 2023), moving models from black boxes to auditable tools.

Real-world deployment depends on engineering for incomplete data, since most patients lack simultaneous multi-omics and imaging profiling; models must handle missing modalities through missingness-aware fusion or robust imputation (Correa-Aguila et al., 2022; Hsu et al., 2025). Liquid biopsy multi-omics represents a pragmatic near-term entry point, sampled repeatedly with lower burden and supported by emerging MRD evidence (Simancas-Racines et al., 2025; Qiu et al., 2025). However, foundation-model embeddings carry substantial computational, storage, and licensing costs that may be prohibitive for resource-limited clinical settings, favoring lighter-weight or federated alternatives where infrastructure is constrained.

Finally, equity and regulatory sustainability must be designed in: uneven sequencing and informatics infrastructure risk favoring high-resource systems (Bonetti et al., 2023; Sabit et al., 2026). A concrete path forward includes biomarker-driven prospective trials, adherence to TRIPOD + AI, and privacy-preserving, multi-institutional data-sharing consortia (Collins et al., 2024; Hsu et al., 2025; Yasmin et al., 2025), converting today’s abundance of discovery-phase signatures into fewer, clinically robust biomarkers with genuine regulatory staying power.

Competing interests: R.S.B. has received honoraria as a speaker: Novartis, AstraZeneca, MSD, Lilly, GSK, Clovis, Seagen, Pfizer, Roche, and Gilead Oncology, Advisory fees: Novartis, Lilly, GSK, Seagen, and Roche, and Non-financial disclosure: ESMO Young Oncologists Committee Member and Scientific Secretary of the Spanish Society of Medical Oncology, all outside the submitted work. C.S. has received travel grants from Eli Lilly, Ipsen, and Novartis, and speaker honoraria from Bayer, all outside the submitted work. All remaining authors declare no conflicts of interest. Transparency Statement: The lead author (D.S.) affirms that this manuscript is an honest, accurate, and transparent account of the study being reported; that no important aspects of the study have been omitted; and that any discrepancies from the study as planned (and, if relevant, registered) have been explained Table 1.
